# Tomato NAC Transcription Factor SlSRN1 Positively Regulates Defense Response against Biotic Stress but Negatively Regulates Abiotic Stress Response

**DOI:** 10.1371/journal.pone.0102067

**Published:** 2014-07-10

**Authors:** Bo Liu, Zhigang Ouyang, Yafen Zhang, Xiaohui Li, Yongbo Hong, Lei Huang, Shixia Liu, Huijuan Zhang, Dayong Li, Fengming Song

**Affiliations:** National Key Laboratory for Rice Biology, Institute of Biotechnology, Zhejiang University, Hangzhou, China; UMBC, United States of America

## Abstract

Biotic and abiotic stresses are major unfavorable factors that affect crop productivity worldwide. NAC proteins comprise a large family of transcription factors that play important roles in plant growth and development as well as in responses to biotic and abiotic stresses. In a virus-induced gene silencing-based screening to identify genes that are involved in defense response against *Botrytis cinerea*, we identified a tomato NAC gene *SlSRN1* (*Solanum lycopersicum* Stress-related NAC1). SlSRN1 is a plasma membrane-localized protein with transactivation activity in yeast. Expression of *SlSRN1* was significantly induced by infection with *B. cinerea* or *Pseudomonas syringae* pv. *tomato* (*Pst*) DC3000, leading to 6–8 folds higher than that in the mock-inoculated plants. Expression of *SlSRN1* was also induced by salicylic acid, jasmonic acid and 1-amino cyclopropane-1-carboxylic acid and by drought stress. Silencing of *SlSRN1* resulted in increased severity of diseases caused by *B. cinerea* and *Pst* DC3000. However, silencing of *SlSRN1* resulted in increased tolerance against oxidative and drought stresses. Furthermore, silencing of *SlSRN1* accelerated accumulation of reactive oxygen species but attenuated expression of defense genes after infection by *B. cinerea*. Our results demonstrate that SlSRN1 is a positive regulator of defense response against *B. cinerea* and *Pst* DC3000 but is a negative regulator for oxidative and drought stress response in tomato.

## Introduction

Plants constantly encounter various biotic (i.e. pathogen infection) and abiotic (i.e. drought, high salinity and extreme temperature conditions) stresses that significantly affect both biomass growth and yield production. However, plants have developed to equip with a sophisticated signaling networks to precisely regulate defense responses against pathogen attack and abiotic stress. Upon perception of the environmental cues, initiation of the signaling network ultimately leads to activation of a large set of genes, which are regulated by different types of transcription factors (TFs). Thus, TFs are critical regulatory factors in modulating the temporal and spatial expression of the genes involved in defense response. During the last decade, numerous TFs belonging to the different families such as NAC, ERF, MYB, WRKY, and bZIP families have been identified to play important roles in regulating plant responses to biotic and abiotic stresses [1]–[5].

The NAC (NAM/ATAF/CUC) TFs are unique plant TFs [6] and comprise a large family with more than 100 members in rice, Arabidopsis, tobacco, potato, soybean, grapevine and poplar [7]–[13]. Structurally, the NAC proteins contain a highly conserved N-terminal DNA-binding domain and a variable C-terminal domain [6]. Recent extensive studies have implicated NAC proteins as important components in different aspects of plant development including formation of boundary cells of the meristem, cell division and expansion, lateral root development, leaf senescence, secondary cell wall biosynthesis, and flowering time (for reviews, see [6], [14]–[18]. In addition, the involvement and function of the NAC proteins in plant responses to biotic and abiotic stresses have been well documented not only in model plants but also in various crop plants (for reviews, see [4], [15], [19], [20]). Thus, it is believed that the NAC proteins can be used as useful functional gene resources for improvement of biotic and abiotic stress tolerance in crops [15], [19], [21].

Accumulating evidence demonstrates that the NAC proteins play critical roles in regulation of plant defense responses against different types of pathogens. The first line of evidence came from the identification of the potato gene *StNAC*, which was induced by pathogen attack [22]. Recent functional studies by altering expression of individual NAC gene through knockout/knockdown and overexpression approaches have identified a number of NAC genes that are involved in defense responses of different plants to pathogen infection. In Arabidopsis, at least eight NAC proteins such as ATAF1, ATAF2, ANAC019, AAC042, ANAC055, ANAC072, CBNAC1 and NTL6 were identified as negative regulators of defense responses against different types of pathogens including *Botrytis cinerea*, *Alternaria brassicicola*, *Fusarium oxysporum* and *Pseudomonas syringae* pv. *tomato* [23]–[31]. In rice, OsNAC6, RIM1, OsNAC4, ONAC122 and ONAC131, were shown to function in regulation of disease resistance against *M. oryzae* and *Rice dwarf virus*, and hypersensitive cell death, respectively [32]–[37]. Similarly, the barley HvNAC6 was demonstrated to increase penetration resistance and promote basal resistance against virulent *Blumeria graminis* f. sp. *hordei*, respectively [38], [39]. Grapevine VvNAC1 and pepper CaNAC1 were also involved in regulation of disease resistance response [40], [41]. Interestingly, the potato and *Nicotiana benthamiana* NTP1 and NTP2 were found to be targeted by an RxLR effector Pi03192 from *Phytophthora infestans* [42]. Thus, it is clear that the NAC proteins participate in many aspects of plants-pathogen interactions, acting as regulators of immune responses or as targets of pathogen effectors.

Recent functional analyses have also provided direct evidence supporting that the NAC proteins function as important components in complex signaling progresses during plant abiotic stress responses (for reviews, see [4], [15], [19], [20]). A number of NAC proteins have been shown to play important roles in plant tolerance to drought and salinity stress. Such NAC proteins include the Arabidopsis ANAC019, ANAC055, ANAC072, ANAC096, ANAC2, ATAF1, ATAF2 and RD26 [23], [43]–[47], rice SNAC1/OsNAC1, OsNAC5, SNAC2/OsNAC6, OsNAC09, OsNAC045, OsNAC052 and OsNAP [32], [48]–[55], and wheat TaNAC69 [56]. Some of NAC proteins have also been implicated in oxidative, temperature and nutrition stresses, for examples, ANAC013 and NTL4 in oxidative stress [57], [58], ANAC042 and ANAC019 in temperature stress [59], [60], IDEF2 and NAM-B1 in nutrition [61], [62].

To date, several NAC genes have been characterized and shown to be responsive to abiotic stresses and play important roles in development of fruit and compound leaves in tomato [63]–[68]. It was found that expression of SlNAC1 was induced by infection with *P. syringae* [69] and SlNAC1 could interact with tomato leaf curl virus replication accessory protein and enhanced viral replication [70]. In this study, we screened dozens of genes by a VIGS-based approach and found that silencing of a tomato NAC gene, *SlSRN1* (*Solanum lycopersicum* stress-related NAC1), led to increased severity of disease caused by *B. cinerea*. Results from further experiments demonstrate that SlSRN1 plays important roles in defense response against biotic stress and tolerance to oxidative and drought stress in tomato.

## Materials and Methods

### Plant growth and treatments

Tomato (*Solanum lycopersicum*) cv. Suhong 2003 was used in this study. Tomato plants were grown in a growth room at 22°C under a 16 hr light and 8 hr dark regime. For analysis of gene expression in response to defense signaling hormones, 4-week-old tomato plants were treated by foliar spraying with 100 µM MeJA (Sigma-Aldrich, MO, USA), 100 µM 1-amino cyclopropane-1-carboxylic acid (ACC) (Sigma-Aldrich, MO, USA), 1 mM salicylic acid (SA) (Sigma-Aldrich, MO, USA) or water as a control. For analysis of gene expression in responding to pathogen infection, 4-week-old plants were inoculated with spore suspension of *B. cinerea*, bacterial suspension of *Pseudomonas syringae* pv. *tomato* (*Pst*) DC3000 or with same volume of buffer as a mock-inoculation control. For analysis of gene expression in drought stress, four-week-old plants were subjected to drought stress treatment by stopping watering for a period until wilting symptom appeared or watered every two days as controls. Alternatively, fully expanded leaves were detached from four-week-old plants and subjected to drought stress treatment by placing on lab blench or on water-saturated filter papers in Petri dishes as controls. Leaf samples were collected at indicated time points after treatment or inoculation and stored at –80°C until use.

### Cloning of *SlSRN1* and bioinformatics analysis

Extraction of total RNA using Trizol reagent (Takara, Dalian, China) was performed according to the manufacturer’s instructions. First-strand cDNA synthesis was performed using the AMV-reverse transcriptase (Takara, Dalian, China) using oligo d(T) primer according to the manufacturer’s instructions. The full-length cDNA of *SlSRN1* was PCR amplified using gene-specific primers SlSRN1-orf-1F (5′-ATG AAG ATG TTT GAG TTA TCT GAT-3′) and SlSRN1-orf-1R (5′-TGG CAA GAT GCC AAA TGA TAG AAC A-3′). The PCR product was cloned into pMD19-T vector (Takara, Dalian, China) and confirmed by sequencing. Similarity searches of nucleotide and amino acid sequences were carried out using BLAST program at the NCBI GenBank database (http://www.ncbi.nlm.nih.gov/BLAST/). Sequence alignment and phylogenetic tree construction were performed by ClustalW method using MegaAlign program in LaserGene software.

### Subcellular localization

The coding sequence of *SlSRN1* was PCR amplified using primers SlSRN1-gfp-1F (5′-AGT GGA TCC ATG AAG ATG TTT GAG TTA TCT GAT TC-3′, a *BamHI* site underlined) and SlSRN1-gfp-1R (5′-GCG TCT AGA TTA AGA GGA TAT GGG TCT CCT-3′, an *XbaI* site underlined). The PCR product was cloned into pFGC-eGFP vector to yield pFGC-SlSRN1 construct. The recombinant plasmid pFGC-SlSRN1 and pFGC-eGFP (as a control) were introduced into onion epidermal cells by the particle bombardment method. Particle bombardment was performed with a PDS-1000 (Bio-Rad, Hercules, CA, USA) according to the manufacturer’s instructions, using gold particles coated with plasmid DNA under a slight vacuum at a helium pressure of 1100 psi. After bombardment, the onion peels were incubated with liquid Murashige-Skoog (MS) medium for 24 hr and GFP was detected with a confocal laser scanning microscope (Zeiss LSM 510 META; argon laser excitation wavelength, 488 nm).

### Transcription activation assay in yeast

The coding sequence of *SlSRN1* was PCR amplified using primers SlSRN1-TA-1F (5′-ATG GTC GAC ATG AAG ATG TTT GAG TTA TCT GAT TC-3′, a *Sal*I site underlined) and SlSRN1-TA-1R (5′-CGA CTG CAG TTA AGA GGA TAT GGG TCT CCT-3′, a *Pst*I site underlined). The resulting PCR products were digested with *Sal*I/*Pst*I and cloned into pBD-GAL4Cam vector, yielding plasmid pBD-SlSRN1. The plasmid pBD-SlSRN1 and pBD empty vector (as a negative control) were transformed into yeast strain AH109. The transformed yeast was cultivated on the SD/Trp^−^ and SD/Trp^−^His^−^ medium for 3 days at 28°C, followed by addition of x-α-gal. The transactivation activity of the fusion protein was evaluated according to the growth situation and production of blue pigments after the addition of x-α-gal of the transformed yeast cells on SD/Trp^−^His^−^ medium.

### Construction of VIGS vectors

For construction of VIGS vectors, a 372 bp fragment of *SlSRN1* was amplified with a pair of gene-specific primers SlSRN1-vigs-1F (5′-GCT GAA TTC AAG AGT GGC TCC GGG CCT AAG-3′, a *Eco*RI site underlined) and SlSRN1-vigs-1R (5′-ATA CTC GAG TGC CTC ATG CAA CTG TCG CT –3′, an *Xho*I site underlined) and cloned into pYL156, yielding TRV-SlSRN1 construct. For construction of TRV-GUS, a 396 bp fragment of the GUS gene was amplified with primers GUS-vigs-1F (5′-CGG TCT AGA ACC TGG GTG GAC GAT ATC AC-3′, an *Xba*I site underlined) and GUS-vigs-1R (5′-CGG GGA TCC GTG CAC CATC AGC ACG TTA T-3′, a *Bam*HI site underlined) and cloned into pYL156, yielding TRV-GUS construct. The recombinant plasmids TRV-SlSRN1 and TRV-GUS were transformed into *A. tumefaciens* strain GV3101 by electroporation.

### Agroinfiltration for VIGS and transient expression

Agrobacteria carrying TRV-SlSRN1, TRV-GUS, pFGC-SlSRN1 or pFGC-eGFP were grown in YEP liquid medium with 50 µg/mL kanamycin, 50 µg/mL rifampicin and 25 µg/mL gentamicin to OD_600_ = 0.8∼1.0. Cells were centrifuged and resuspended in infiltration MES buffer (pH5.7, 10 mM MES, 10 mM MgCl_2_ and 150 µM acetosyringone). For VIGS agroinfiltration, agrobacteria carrying TRV-GUS or TRV-SlSRN1 were mixed with agrobacteria carrying TRV1 in a ratio of 1∶1 and maintained at OD_600_ = 1.5 for 3 hr at room temperature. The mixed agrobacteria suspension was infiltrated into the abaxial surface of the 2-week-old seedlings using a 1 mL needleless syringe. Efficiency of the VIGS protocol was evaluated using phytoene desaturase (PDS) gene as a marker of silencing in tomato plants according to Liu et al. [71]. The VIGS-infiltrated plants were allowed to grow for three weeks under same condition as mentioned above and then used for all experiments. For transient expression agroinfiltration, agrobacteria carrying pFGC-SlSRN1 or pFGC-eGFP empty vector were infiltrated into leaves of 4-week-old plants using 1 mL needleless syringes. Leaf samples were collected 2 days after agroinfiltration for analyzing the expression level of *SlSRN1* and were used for disease assays.

### Disease assays

Inoculation of tomato plants with *B. cinerea* was carried out as described previously [72]. Briefly, spores were collected from 10-day-old *B. cinerea* cultures and resuspended in 4% maltose and 1% peptone buffer to a concentration of 1×10^5^ spores/mL. Detached fully expanded leaves were inoculated by drop inoculation method according to a previously reported procedure [72]. For whole plant inoculation, 4-week-old plants were sprayed with spore suspension or buffer as mock inoculation control. The inoculated leaves or plants were covered with a transparent plastic film and kept in a growth chamber with similar conditions as for plant growth. Diameters of each lesion were recorded 4 days post inoculation (dpi). Leaves from at least ten individual plants were used in each independent experiment.

*Pst* DC3000 grown overnight in King’s B liquid medium containing 25 µg/mL rifampicin were diluted and re-grown to OD_600_ = ∼1.0. Bacteria were collected and resuspended in 10 mM MgCl_2_ to OD_600_ = 0.0002. Four-week-old plants were vacuum infiltrated with bacteria suspensions and then kept in a growth chamber with high humidity. For measurement of bacterial growth curve, leaf punches from six individual plants were surface sterilized in 70% ethanol for 10 sec, homogenized in 200 µL of 10 mM MgCl_2_, diluted in 10 mM MgCl_2_, and plated on KB agar plates containing 100 µg/mL rifampicin.

### Abiotic stress assays

For oxidative stress assays, fully expanded leaves from TRV-SlSRN1- or TRV-GUS-infiltrated plants were collected 2 weeks after VIGS infiltration and rinsed with sterile distilled water. Leaf discs (13 mm in diameter) were made by a hole puncher from at least 6 individual plants for each experiment and were incubated in 1/2 MS buffer supplemented with 20 mM H_2_O_2_ or without H_2_O_2_ (as a control) for 3 days under illumination condition at moderate light intensity (200 µmol m^−2 ^s^−1^). Measurement of chlorophyll content was performed as described before [73] and the content of chlorophyll was calculated according to the formula Chl (A+B) = 5.24A_664_+22.24A_648_. In drought stress assays, the TRV-SlSRN1- or TRV-GUS-infiltrated plants were allowed for further growth with normal watering regime for 2 weeks after VIGS infiltration and then were subjected to drought stress by stopping watering for a certain period of time until the wilting symptoms were obvious. Measurement of the relative water content (RWC) in leaves was performed as described before [74]. Fully expanded leaves from 6 individual plants were detached to measure the leaf fresh weight (W_F_), turgid leaf weight (W_T_), and dry weights (W_D_) and relative water contents (RWC) were calculated from the equation RWC (%) = (W_F_−W_D_)/(W_T_−W_D_)×100% [74]. All experiments were repeated independently for three times.

### Quantitative RT-PCR analysis of gene expression

Silencing efficiency of *SlSRN1* in TRV-SlSRN1-infiltrated plants, expression of *SlSRN1* and defense genes were analyzed by qRT-PCR. Tomato *SlActin* was used as a reference gene with primers of SlActin-1F (5′-GAA ATA GCA TAA GAT GGC AGA CG-3′) and SlActin-1R (5′-ATA CCC ACC ATC ACA CCA GTA T-3′). Primers for *SlSRN1* and other defense genes are as follows: SlSRN1-q-1F, 5′-GCA TGA GGC ACT AGA AGT CAC ATC T-3′; SlSRN1-q-1R, 5′-CCA AGA AGG TCA TCC ATC TCC AGA A-3′; SlPR1a-q-1F, 5′-TCT TGT GAG GCC CAA AAT TC-3′; SlPR1a-q-1R, 5′-ATA GTC TGG CCT CTC GGA CA-3′; SlPR1b-q-1F, 5′-CCA AGA CTA TCT TGC GGT TC-3′; SlPR1b-q-1R, 5′-GAA CCT AAG CCA CGA TAC CA-3′; SlPR5-q-1F, 5′-AAT TGC AAT TTTA ATG GTG C-3′; SlPR5-q-1R, 5′-TAG CAG ACC GTT TAA GAT GC-3′; SlTPK1b-q-1F, 5′-CTG TTA GCA TAG ATG GTG GTG AT-3′; SlTPK1b-q-1R, 5′-CGA AAG TTC CTA GTG GCT GTT TT-3′; SlAREB1-q-1F, 5′-GTG GTG GGA AGG ATG GAA ATA-3′; SlAREB1-q-1R, 5′-CTC TCA CAA CTC CAG CTC TAAC-3′; SGN-213276-q-1F, 5′-GTC AAA CAC TGG AAA GCA TGA A-3′; SGN-213276-q-1R, 5′-AGC TGC TCC ACT TGT CTT ATC-3′. Relative expression was calculated using 2^–ΔΔCT^ method. The experiments were repeated independently with three biological replicates using SYBR Green PCR master mix kit (Takara, Dalian, China) in a CFX96 real-time PCR detection system (Bio-Rad, Hercules, CA, USA) according to the manufacturer’s instructions.

### Detection of reactive oxygen species

Detection of H_2_O_2_ and superoxide anion (O_2_^−^) in leaf tissues was performed by 3,3-diaminobenzidine (DAB) and nitroblue tetrazolium (NBT) staining, respectively, according to the methods described previously [75], [76]. Leaf samples were collected from inoculated plants at 0 and 24 hr after inoculation with *B. cinerea* and dipped into DAB solution (1 mg/mL, pH 3.8) for staining of H_2_O_2_, or in NBT solution (1 mg/mL NBT in 10 mM NaN_3_ and 10 mM phosphate buffer, pH 7.8) for staining superoxide anion. Accumulation of H_2_O_2_ and superoxide anion in leaves was visualized using a digital camera.

## Results

### Characterization of SlSRN1

**Figure 1:**
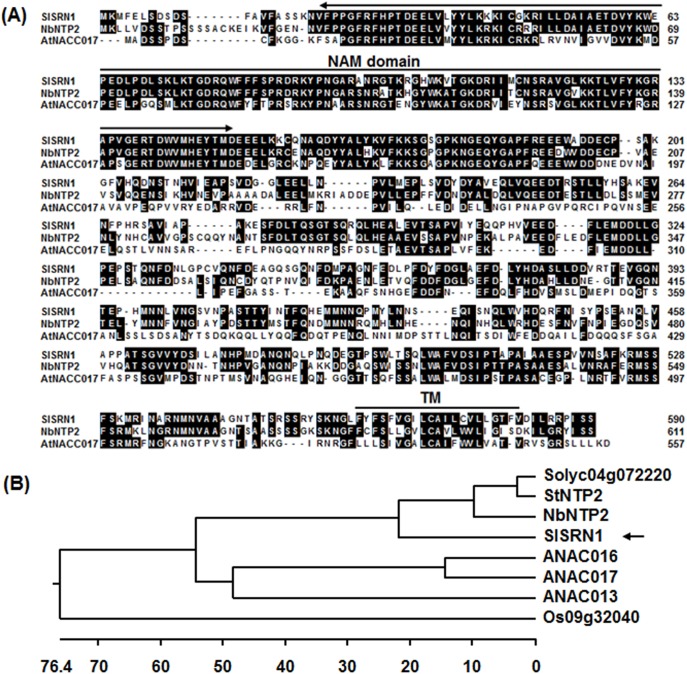
Sequence alignment and phylogenetic tree analysis of SlSRN1 with other plant NAC proteins. A. Sequence alignment of SlSRN1 with NbNTP2 and AtNAC017. The numbers on the left indicate amino acid positions of the proteins used. Shared amino acid residues are shown in black background. Conserved NAC domain and putative transmembrane motif are indicated. B. Phylogenetic tree analysis of SlSRN1 with other plant NAC proteins. Phylogenetic tree was constructed by neighbour-joining method using MEGA program version 6.05. SlSRN1 in the tree is indicated by an arrow. Plant NAC proteins used and their GenBank accessions are as follows: Arabidopsis AtNAC017 (AAK32826), AtNAC016 (AAD39614) and AtNAC013 (AEE31534), *Nicotiana benthamiana* NbNTP2 (AGY49287), potato StNTP2 (AGY49285) a tomato predicted NAC (Solyc04g072220) and rice Os09g32040 (BAF25461).

To explore the molecular basis of defense response in tomato against necrotrophic fungal pathogens, we performed VIGS-based screening to identify genes that are involved in defense response to *B. cincrea*. In these VIGS fast screenings, dozens of genes were chosen and examined for altered phenotype of disease caused by *B. cinerea*. It was observed that knockdown of a gene encoding for a NAC transcription factor led to increased severity of disease caused by *B. cinerea*. This putative NAC gene, designated as *SlSRN1* for *Solanum lycopersicum* stress-related NAC1, was chosen for further study. A full-length cDNA, SGN-U320122, and two partial cDNAs, SGN-U585287 and SGN-U587626, for *SlSRN1* were identified by blast searches against the tomato genomic database (http://solgenomics.net/). The *SlSRN1* gene corresponds to the predicted locus Solyc12g056790 on chromosome 12 and comprises 2 introns and 3 exons. After cloning and confirmation of the sequence, the full-length cDNA of *SlSRN1* is 1987 bp with an open reading frame of 1773 bp, which encodes a protein of 590 residues with a calculated molecular mass of 66.5 kDa and a theoretical pI of 4.8. The SlSRN1 protein contains a conserved NAM domain (residues 22–150) at N-terminal and a typical α helix transmembrane motif (561-FYFSFVGILCAILCVLLGTFV-581, HyPhob = 0.819) at C-terminal (Fig. 1A). Phylogenetic tree analysis revealed that SlSRN1 shows 69–72% of identity to NbNPT2 [42], potato StNTP2 [42]) and another predicted tomato NAC protein Solyc04g072220, and 46–50% of identity to Arabidopsis ANAC017 [77], ANAC016 [78] and ANAC013 [58] (Fig. 1B).

### SlSRN1 is localized on the plasma membrane and has transactivation activity

**Figure 2:**
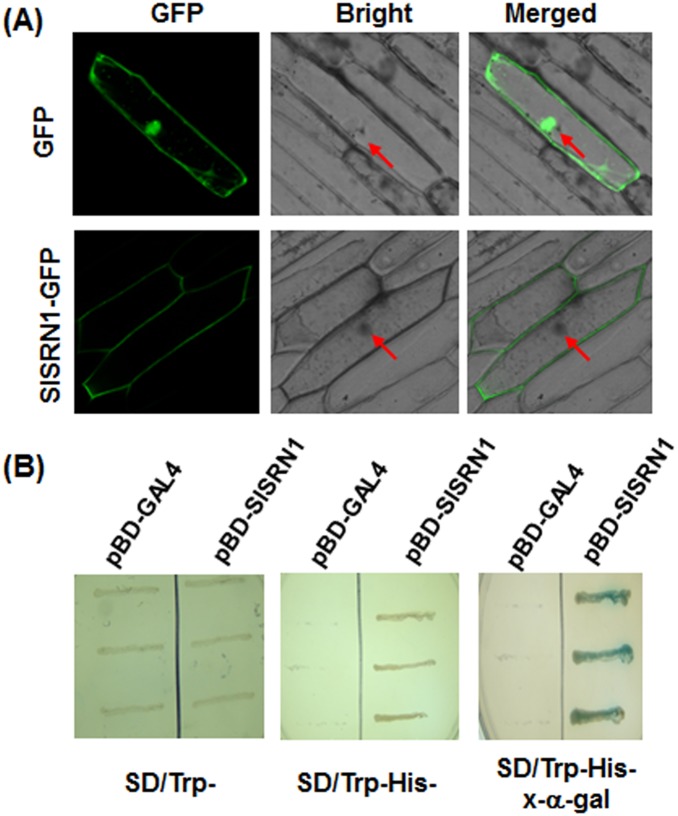
Subcellular localization and transactivation activity of SlSRN1. A. Subcellular localization of SlSRN1 when transiently expressed in onion epidermal cells. Onion epidermal cells were transiently transformed with either control GFP vector (upper) or SlSRN1-GFP construct (lower) by particle bombardment. The subcellular localization of the SlSRN1-GFP fusion protein and GFP alone were viewed at 24 hr after bombardment under a confocal laser microscopy in dark field for green fluorescence (*left*), in white field for the morphology of the cell (*middle*), and in combination (*right*), respectively. Red arrows indicate the nucleuses of the onion epidermal cells. B. Transactivation activity of SlSRN1 in yeast. Yeasts carrying pBD-SlSRN1 or pBD empty vector (as a negative control) were streaked on the SD/Trp^−^ plates (*left*) or SD/Trp^−^His^−^ plates (*middle*) for 3 days at 28°C. The x-α-gal was added to the SD/Trp^−^His^−^ plates and kept at 28°C for 6 hr (*right*).

To determine the subcellular localization of SlSRN1, a SlSRN1-GFP fusion construct was generated and introduced into onion epidermal cells by particle bombardment. When transiently expressed, the SlSRN1-GFP fusion protein was localized exclusively on the plasma membrane of onion epidermal cells but not in the nucleus, while the GFP fluorescence was observed throughout the entire cytoplasm and the nucleus without specific compartment localization (Fig. 2A). This result indicates that the SlSRN1 is localized to the plasma membrane of cells.

Transactivation activity of SlSRN1 was examined using a yeast assay system. As shown in Fig. 2B, both yeast transformants carrying pBD-SlSRN1 and pBD vector grew well on SD/Trp^−^ medium. However, only yeast transformants containing pBD-SlSRN1 were able to grow on the SD/Trp^−^His^−^ medium and produced a blue pigment after the addition of x-α-gal, showing a β-galactosidase activity, whereas transformants containing the pBD empty vector did not. These results indicate that SlSRN1 has transactivation activity in yeast cells.

### Induced expression of *SlSRN1* by pathogens and defense signaling hormones

**Figure 3:**
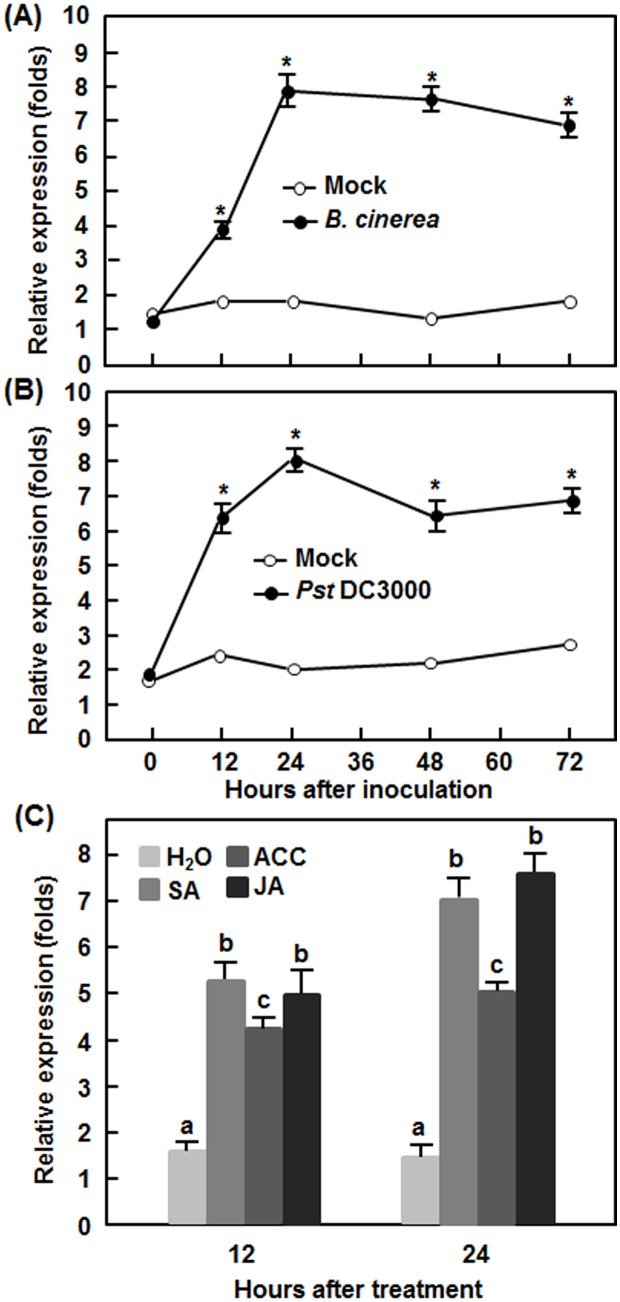
Expression of *SlSRN1* in responses to pathogen infection and treatments with defense signaling hormones. Four-week-old tomato seedlings were inoculated by spore suspension of *B. cinerea* (A), bacterial suspension of *Pst* DC3000 (B) or similar volume of buffer solution as mock-inoculation control or treated by foliar spraying with 1 mM SA, 100 µM MeJA, 100 µM ACC solutions or sterilized distill water as a control (C). Leaf samples were collected at different time points after inoculation or treatment as indicated. Total RNA was extracted and used for qRT-PCR analysis. Data presented are the means ± SD from three independent experiments and different letters above the columns indicate significant differences at *p*<0.05 level.

To explore the possible involvement of *SlSRN1* in tomato disease resistance response, we analyzed the expression dynamics of *SlSRN1* in response to infection by different types of pathogens and treatment with defense signaling hormones. The expression of *SlSRN1* in mock-inoculated plants maintained unchanged in *B. cinerea*-inoculated plants during the experimental period (Fig. 3A). However, the expression level of *SlSRN1* started to increase at 12 hr post inoculation (hpi), peaked at 24 hpi and maintained at a very high level until 72 hpi, showing increases of 3–8 folds over that in mock-inoculated plants during the experimental period (Fig. 3A). Similar expression dynamic of SlSRN1 was observed in plants after infection with *Pst* DC3000 (Fig. 3B). In *Pst* DC3000-inoculated plants, the expression level of *SlSRN1* increased at 12 hpi, peaked at 24 hpi and maintained at a relatively high level until 72 hpi, giving increases of 6–8 folds over that in mock-inoculated plants (Fig. 3B). In defense signaling hormone-treated plants, expression of *SlSRN1* was induced by SA, JA and ACC, showing increases of approximately 4–7 folds over that in control plants at 12 and 24 hr after treatment (Fig. 3C). These results indicate that expression of *SlSRN1* can be induced by infection of *B. cinerea* and *Pst* DC3000 and by treatment with defense signaling hormones.

### SlSRN1 is required for resistance against *B. cinerea*

**Figure 4:**
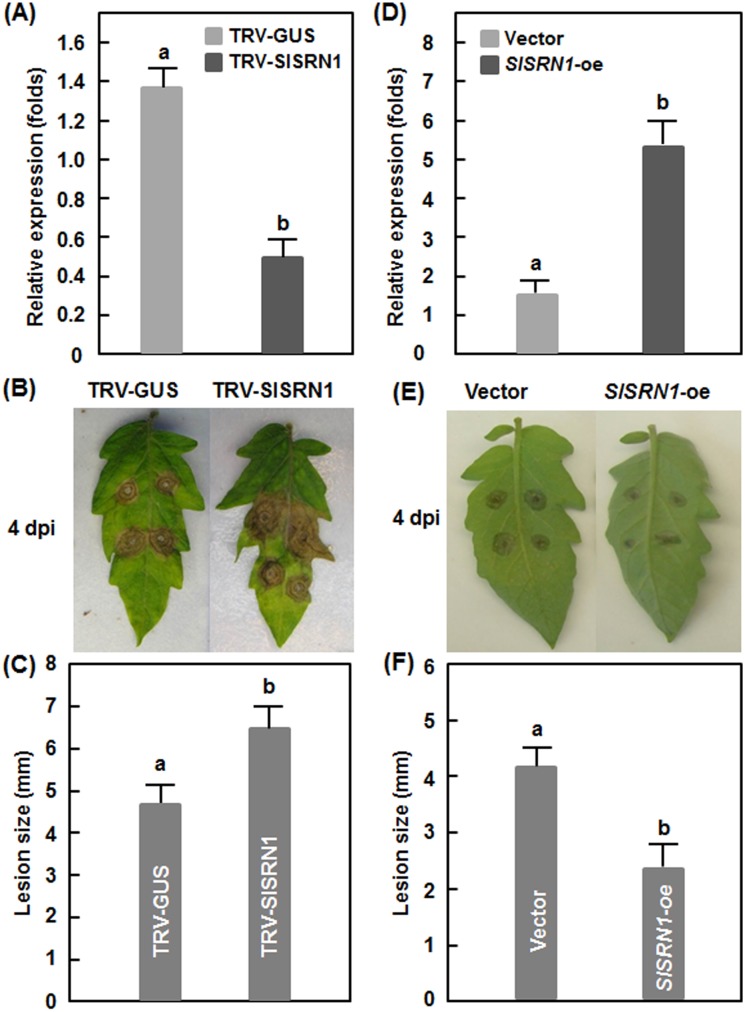
*SlSRN1* positively regulates resistance response against *B. cinerea*. A–C. Silencing of *SlSRN1* led to enhanced susceptibility to *B. cinerea*. A. Silencing efficiency in TRV-SlSRN1-infiltrated tomato plants. Two-week-old seedlings were infiltrated with agrobacteria carrying TRV-SlSRN1 or TRV-GUS and leaf samples were collected 4 weeks after VIGS treatment. The transcript of *SlSRN1* was analysed by qRT-PCR. B and C. Disease phenotype and lesion size on detached leaves of TRV-SlSRN1 or TRV-GUS-infiltrated plants after inoculation with *B. cinerea*, respectively. D-E. Transient overexpression of *SlSRN1* resulted in increased resistance to *B. cinerea*. D. Expression of *SlSRN1* in pFGC-SlSRN1- or pFGC-eGFP-infiltrated plants. Leaves of 3-week-old seedlings were infiltrated with agrobacteria carrying pFGC-SlSRN1- or pFGC-eGFP vector and leaf samples were collected 3 days after infiltration. E and F. Disease phenotype and lesion size on detached leaves of pFGC-SlSRN1- or pFGC-eGFP-infiltrated plants after inoculation with *B. cinerea*, respectively. Data presented are the means ± SD from three independent experiments and different letters above the columns indicate significant differences at *p*<0.05 level.

To explore the possible function of *SlSRN1* in disease resistance, we used the TRV-based gene silencing system [71] to knockdown the expression level of *SlSRN1* in tomato plants and compared the phenotype between the silenced and the control plants after infection with *B. cinerea* or *Pst* DC3000. For this purpose, we made TRV-mediated VIGS constructs for *SlSRN1* genes and performed standard VIGS procedure on two-week-old tomato seedlings. Only when the efficiency of the VIGS protocols was >90%, judged based on the appearance of bleaching phenotype in the pTRV-PDS-infiltrated plants, the TRV-SlSRN1-infiltrated plants in the same batch were used for various experiments three weeks after VIGS infiltration. The silencing efficiency for *SlSRN1* under our experimental condition was ∼70% (Fig. 4A), as examined by qRT-PCR analysis of the transcript level of *SlSRN1* in the TRV-SlSRN1-infiltrated plants and compared with that in the TRV-GUS-infiltrated negative control plants.

We first examined the disease phenotype of the TRV-SlSRN1-infiltrated plants after inoculation with *B. cinerea* using a detached leaf inoculation assay. Under our disease assay conditions, typical disease symptom, e.g. necrotic lesions, was observed in the leaves from the TRV-SlSRN1- and TRV-GUS-infiltrated plants 2 dpi but the lesions in the leaves from the TRV-SlSRN1-infiltrated plants expanded much rapidly and were larger than those in the TRV-GUS-infiltrated plants (Fig. 4B). At 4 dpi, the lesion size in the leaves from the TRV-SlSRN1-infiltrated plants showed an average of 6.5 mm, giving an increase of 38% over that in the TRV-GUS-infiltrated plants (average of 4.7 mm for lesion size) (Fig. 4C). Meanwhile, we also explored whether transient expression of *SlSRN1* in tomato leaves can confer an increased resistance against *B. cinerea*. As shown in Fig. 4D, the expression level of *SlSRN1* in leaves of plants infiltrated with agrobacteria carrying pFGC-SlSRN1 construct increase significantly at 2 days after infiltration, leading to approximately 4 times higher over that in the control plants infiltrated with agrobacteria carrying pFGC-eGFP vector only. Disease assays revealed that the lesions on leaves of the pFGC-SlSRN1-infiltrated plants were smaller than those on leaves of the pFGC-eGFP-infiltrated plants (Fig. 4E), resulting in a reduction of 40% in size (Fig. 4F). These data indicate that silencing of *SlSRN1* resulted in increased disease while transient overexpression led to reduced disease caused by *B. cinerea*, demonstrating that SlSRN1 plays an important role in resistance against *B. cinerea*.

### Silencing of *SlSRN1* resulted in increased disease caused by *Pst* DC3000

**Figure 5:**
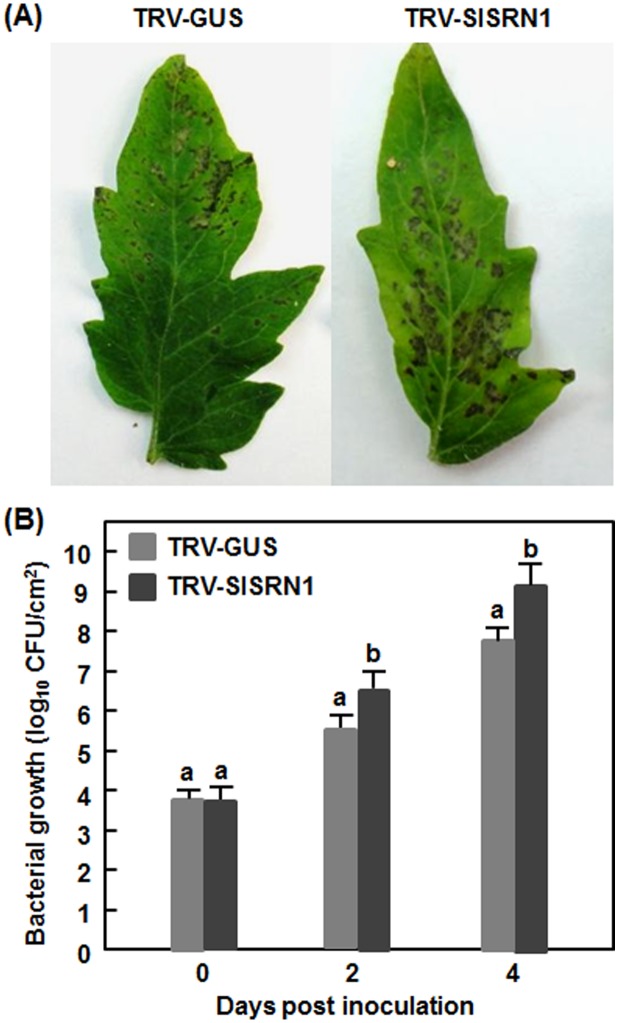
Silencing of *SlSRN1* resulted in increased susceptibility to *P. syringae* pv. *tomato* DC3000. Two-week-old seedlings were infiltrated with agrobacteria carrying TRV-SlSRN1 or TRV-GUS and were inoculated with *Pst* DC3000 two weeks after VIGS infiltration. Disease phenotype (A) and bacterial population (B) in leaves of TRV-SlSRN1- or TRV-GUS-infiltrated plants were recorded. Data presented are the means ± SD from three independent experiments and different letters above the columns indicate significant differences at *p*<0.05 level.

We further examined whether SlSRN1 is involved in resistance against *Pst* DC3000. In out experiments, necrotic lesions were observed in the inoculated leaves of the TRV-SlSRN1- and TRV-GUS-infiltrated plants; however, the lesions on leaves of the TRV-SlSRN1-infiltrated plants were larger than those in the TRV-GUS-infiltrated plants (Fig. 5A). At 2 dpi, the bacterial population in the inoculated leaves of the TRV-SlSRN1-infiltrated plants showed a 10-fold higher over that in the TRV-GUS-infiltrated plants (Fig. 5B). At 4 dpi, the bacterial population in inoculated leaves of the TRV-SlSRN1-infiltrated plants was measured to be 1.26×10^9^ cfu/cm^2^, showing a 16-fold increase in bacterial growth relative to that in the TRV-GUS-infiltrated plants (7.9×10^7^ cfu/cm^2^) (Fig. 5B). These results indicate that silencing of *SlSRN1* resulted in enhanced disease severity and increased bacterial growth, implying the requirement of *SlSRN1* for resistance against *Pst* DC3000.

### Silencing of *SlSRN1* affects defense response against *B. cinerea*

**Figure 6:**
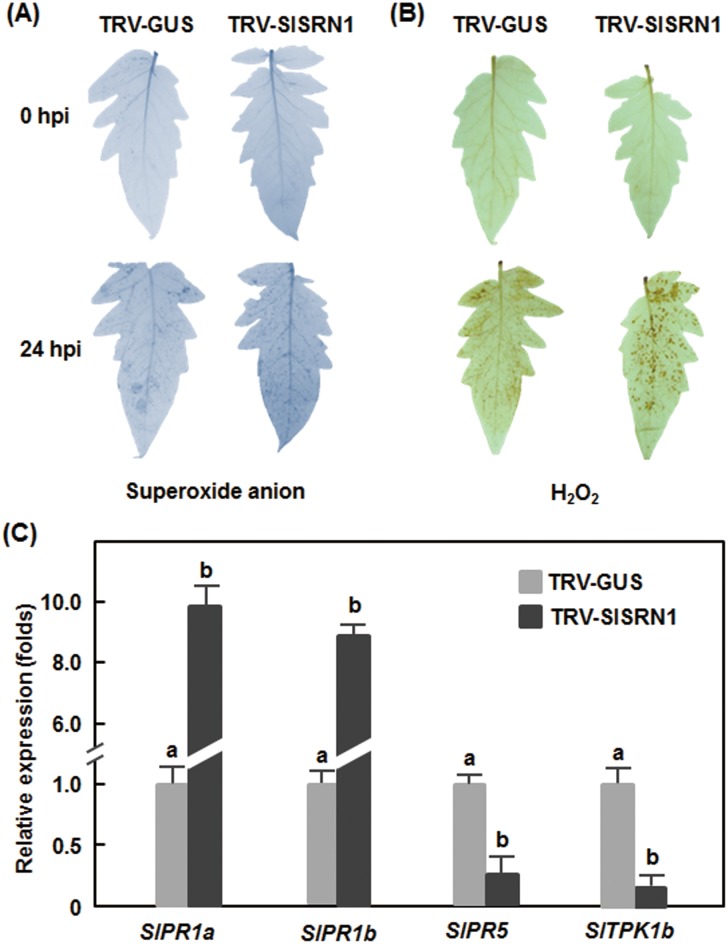
Altered generation of reactive oxygen species and expression of defense genes in *SlSRN1*-silenced plants after infection with *B. cinerea.* Two-week-old seedlings were infiltrated with agrobacteria carrying TRV-SlSRN1 or TRV-GUS and were inoculated by spraying with spore suspension of *B. cinerea* at 2 weeks after VIGS infiltration. Leaf samples were collected at 0 (as controls) and 24 hr after inoculation for detection of reactive oxygen species and analyses of defense gene expression. A and B. Detection of H_2_O_2_ and superoxide anion by DAB and NBT staining, respectively. Representative stained leaves are shown and the experiments were repeated twice with similar results. C. Expression of defense genes after infection with *B. cinerea*. Relative expression levels were calculated by comparing with the corresponding values at 0 h after treatment (as a control). At least 6 leaves from 6 individual silenced or control plants were used for each experiment. Data presented are the means ± SD from three independent experiments and different letters above the columns indicate significant differences at *p*<0.05 level.

We first examined whether silencing of *SlSRN1* affects accumulation of reactive oxygen species (ROS) upon infection of *B. cinerea*. No significant accumulation of H_2_O_2_ and superoxide anion was detected in leaves of the TRV-SlSRN1- and TRV-GUS-infiltrated plants at 0 hr after inoculation (Fig. 6A and B), indicating that silencing of *SlSRN1* did not affect accumulation of ROS in tomato plants. At 24 hr after inoculation with *B. cinerea*, accumulation of superoxide anion and H_2_O_2_ in leaves of the TRV-SlSRN1-infiltrated plants showed significant increases as compared with those in the TRV-GUS-infiltrated plants (Fig. 6A and B), especially the increase of H_2_O_2_ accumulation in leaves of the TRV-SlSRN1-infiltrated plants (Fig. 6B).

We further examined the effect of *SlSRN1* silencing on the expression of defense- and signaling-related genes in tomato plants after infection by *B. cinerea*. For this purpose, we analyzed and compared the expression levels of *SlPR1a*, *SlPR1b*, *SlPR5* and *STPK1b* in the TRV-SlSRN1- and TRV-GUS-infiltrated plants after infection by *B. cinerea*. As shown in Fig. 6C, expression levels of *SlPR1a* and *SlPR1b* in the TRV-SlSRN1-infiltrated plants were significantly increased, showing 9–10 folds higher than those in the TRV-GUS-infiltrated plants after infection with *B. cinerea*. However, the expression levels of *SlPR5* and *SlTPK1b* in the TRV-SlSRN1-infiltrated plants were markedly decreased, leading to a reduction of 75–90% as compared with those in the TRV-GUS-infiltrated plants after infection with *B. cinerea* (Fig. 6C). These data indicate that silencing of *SlSRN1* affects the expression of a set of defense- and signaling-related genes upon infection with *B. cinerea*.

### *SlSRN1* is required for tolerance to oxidative and drought stress

**Figure 7:**
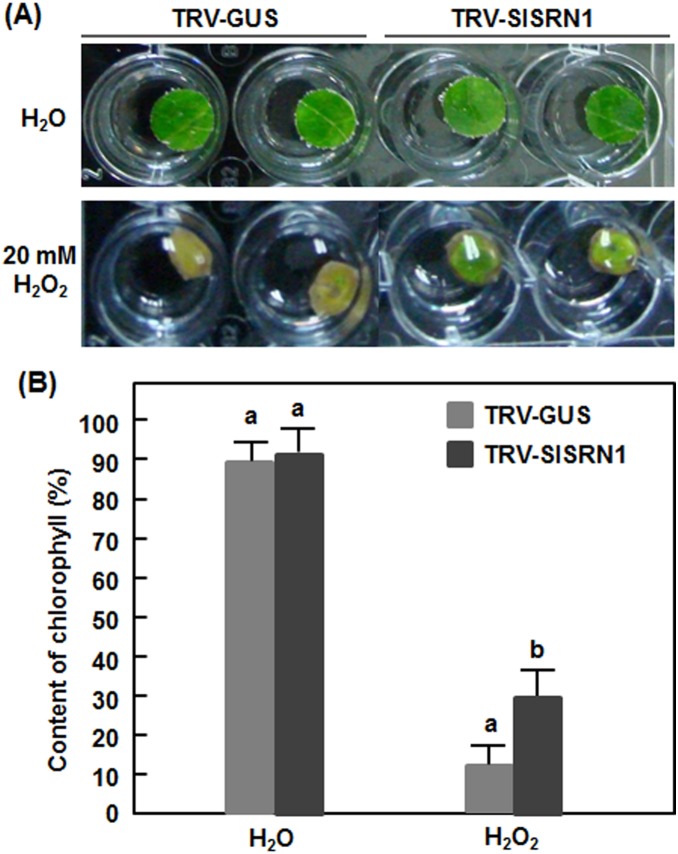
Silencing of *SlSRN1* increased tolerance to oxidative stress. Two-week-old seedlings were infiltrated with agrobacteria carrying TRV-SlSRN1 or TRV-GUS and leaf discs were taken from leaves of TRV-SlSRN1- or TRV-GUS-infiltrated plants at 2 weeks after VIGS infiltration. Leaf discs were soaked in 1/2 MS buffer supplemented with 20 mM H_2_O_2_ or H_2_O (as a control). Phenotype (A) and relative chlorophyll contents (B) in leaf discs from TRV-SlSRN1- or TRV-GUS-infiltrated plants under oxidative stress. Photos and samples for analysis of chlorophyll contents were taken at 5 days after treatment. Data presented in (B) are the means ± SD from three independent experiments and different letters above the columns indicate significant differences at *p*<0.05 level.

To explore whether SlSRN1 has a function in abiotic stress response, we analyzed and compared the oxidative stress tolerance and drought tolerance of the TRV-SlSRN1- and TRV-GUS-infiltrated plants. In oxidative stress assays, leaf discs from leaves of the TRV-SlSRN1- and TRV-GUS-infiltrated plants were treated in H_2_O_2_ solution as an artificial oxidative stress condition. During 5 days of the experimental period, no significant phenotype appeared on the leaf discs from the TRV-SlSRN1- and TRV-GUS-infiltrated plants without H_2_O_2_ treatment (Fig. 7A). When treated with H_2_O_2_, bleaching and chlorosis symptom were observed in leaf discs from the TRV-SlSRN1- and TRV-GUS-infiltrated plants; however, bleaching and chlorosis symptoms in leaf discs from the TRV-SlSRN1-infiltrated plants were less severe than those of the TRV-GUS-infiltrated plants (Fig. 7A). This observation was further confirmed by measuring chlorophyll contents in leaf discs from the TRV-SlSRN1- and TRV-GUS-infiltrated plants after H_2_O_2_ treatments (Fig. 7B). Without H_2_O_2_ treatment, no significant difference in relative chlorophyll contents in leaf discs from the TRV-SlSRN1- and TRV-GUS-infiltrated plants was observed. However, relative chlorophyll contents in leaf discs from the TRV-SlSRN1- and TRV-GUS-infiltrated plants were dramatically decreased after treatments with H_2_O_2_ (Fig. 7B). Notably, relative chlorophyll contents, measuring approximately 29.5% at 5 days after treatment, in leaf discs from the TRV-SlSRN1-infiltrated plants were significantly higher than that, measuring about 12.2%, from the TRV-GUS-infiltrated plants (Fig. 7B). These results indicate that silencing of *SlSRN1* strengthens oxidative stress tolerance in tomato.

**Figure 8:**
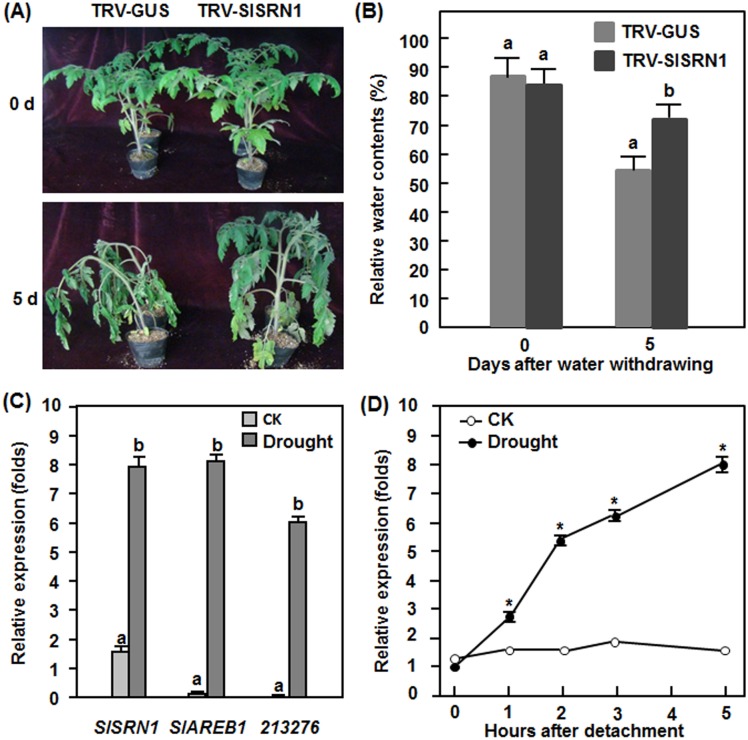
Silencing of *SlSRN1* increased tolerance to drought stress. (A) and (B), Phenotype (A) and relative water contents (B) in leaves from the TRV-SlSRN1- or TRV-GUS-infiltrated plants at 5 days after drought treatment. Two-week-old seedlings were infiltrated with agrobacteria carrying TRV-SlSRN1 or TRV-GUS and allowed for further growth for another 2 weeks. The TRV-SlSRN1- or TRV-GUS-infiltrated plants were treated for drought stress by stopping watering for a period until wilting symptom was appeared. (C) and (D), Expression of *SlSRN1* induced by drought stress. Four-week-old tomato plants were treated for drought stress by stopping watering for a period or watered normally as controls and leaf samples were collected at 7 days after treatment when wilting symptom appeared (C). Fully expanded leaves were detached from four-week-old plants and subjected to drought stress treatment by placing on lab blench or water-saturated filter papers in Petri dishes as a control and samples were collected at different time points as indicated. Total RNA was extracted and used for qRT-PCR analysis. Data presented in (B), (C) and (D) are the means ± SD from three independent experiments and different letters above the columns and the asteriks above the lines indicate significant differences at *p*<0.05 level.

In drought stress assays, the appearance and wilting phenotype in the TRV-SlSRN1-infiltrated plants was less severe than that in the TRV-GUS-infiltrated plants during 7 days of experimental period (Fig. 8A). At the time starting to drought treatment, RWCs in leaves of the TRV-SlSRN1- and TRV-GUS-infiltrated plants were similar, measuring approximately 84–87% (Fig. 8B). At 5 days after drought treatment, RWC in leaves of the TRV-SlSRN1-infiltrated plants (∼72%) was significantly higher than that in the TRV-GUS-infiltrated plants (∼53%), giving an increase of 36% in RWC as compared that in the TRV-GUS-infiltrated plants (Fig. 8B). These data indicate that silencing of *SlSRN1* improves drought stress tolerance in tomato. To further confirm this conclusion, we analyzed the expression of *SlSRN1* during drought stress. In the whole plant assays, the expression of *SlSRN1* was significantly induced by drought stress, leading to a 5-fold increase over that in the control plants (Fig. 8C). This coincided with the up-regulated expression of two previously reported tomato drought-responsive genes, *SlAREB1* [79] and *SGN-213276* [80] (Fig. 8C). Similar expression pattern of *SlSRN1* was also observed in the detached leaf assay. The expression level of *SlSRN1* in drought stress-treated leaves started to increase at 1 hr after treatment and increased gradually during an experimental period of 5 hr (Fig. 8D) whereas the expression of *SlSRN1* in water-saturated leaves remained unchanged during the experimental period (Fig. 8D). These data indicate that *SlSRN1* is a drought stress-responsive gene in tomato.

## Discussion

Regulation of gene expression at the transcriptional level is critical to activate effective defense responses upon biotic and abiotic stresses. The NAC proteins comprise a large family of transcription factors with more than 100 members in plant species whose genomes have been completely sequenced so far. Recently, genome-wide bioinformatics analysis identified 110 *StNAC* genes in potato encoding for 136 proteins, including 14 membrane-bound NAC proteins [12]. It is possible that there are similar number of NAC genes and proteins in tomato. In this regarding, only a few of the tomato NAC proteins have been studied in detail for their biological functions, e.g. SlNAC4 in fruit ripening and carotenoid accumulation [68], SlNAM2 in flower-boundary morphogenesis [67], SlNAC1 in chilling tolerance [66] and in enhancing viral replication via interaction with replication accessory protein [70], GOBLET in determining leaflet boundaries of compound leaves [63]. The present study identified a pathogen-responsive tomato NAC gene *SlSRN1* and demonstrated that SlSRN1 positively regulates defense response against biotic stress but negatively regulates tolerance to oxidative and drought stresses. Our findings from functional characterization of SlSRN1 expand the list of NAC proteins with known biological functions in the tomato NAC family.

The SlSRN1 protein contains a typical NAC domain at N-terminal and a putative transmembrane motif at C-terminal (Fig. 1A) and shows the highest levels of identity to NbNPT2 and potato StNTP2 [42] and Arabidopsis ANAC017 [77], ANAC016 [78] and ANAC013 [58]. The presence of a transmembrane motif and high level of sequence identity to recently functionally characterized NAC proteins suggest that SlSRN1 belongs to the membrane-bound transcription factors [81]. The ANAC017, NbNTP2 and StNPT2 were shown to target endoplasmic reticulum membrane [42], [77]. Similarly, we also observed that the SlSRN1 was targeted to the plasma membrane when transiently expressed in onion epidermal cells (Fig. 2A). In our study, the SlSRN1 has transactivation activity in yeast cells (Fig. 2B), indicating that SlSRN1 is a transcriptional activator. Similarly, the ANAC013, ANAC016 and ANAC017, three closest homologs of SlSRN1 in Arabidopsis, were demonstrated to bind to *cis*-elements in promoters of their downstream target genes and initiate the expression of these targeting genes [58], [77], [78]. Recently, it was shown that the StNTP2 protein was released from the ER membrane after treatment with *P. infestans* culture filtrate, which can induce rapidly the expression of *StNPT2*, and accumulated in the nucleus [42]. Thus, it is likely that SlSRN1 and its homologs like StNTP2 have a dynamic subcellular localization to exert their biochemical function upon different environmental stress signals. Further identification of the SlSRN1-regulated target genes will provide direct evidence suggesting the mechanism of SlSRN1 in regulation of downstream gene expression.

Our VIGS-based experimental results demonstrate that SlSRN1 is a positive regulator of defense response against biotic stress including *B. cinerea* and *Pst* DC3000. Expression of *SlSRN1* was dramatically induced by *B. cinerea* and *Pst* DC3000 within the early stage of infection (12–72 hr after inoculation), indicating that *SlSRN1* is an early pathogen-responsive gene (Fig. 3). This is similar to the expression pattern of *StNTP2* in response to treatment with culture filtrate of *P. infestans*, in which rapid induction of *StNTP2* expression was observed at 3 hr after treatment [42]. Another, expression of *SlSRN1* was induced significantly by SA, JA and ACC, three well-known defense signaling hormones (Fig. 3C). Collectively, pathogen- and defense signaling hormone-inducible expression features imply that *SlSRN1* is positively involved in defense response against pathogen infection. Direct evidence supporting a role for SlSRN1 in disease resistance response came from our VIGS-based experiments. In our study, we found that silencing of *SlSRN1* resulted in increased severity of diseases caused by both *B. cinerea* and *Pst* DC3000 whereas transient expression of *SlSRN1* led to decreased severity of disease by *B. cinerea* (Fig. 4 and 5). This is in line with the observations that silencing of *NbNTP2*, showing high level of identity to SlSRN1 (Fig. 1B), markedly increased severity of disease caused by *P. infestans* in *N. benthamiana* [42]. Therefore, we concluded that *SlSRN1* is required for disease resistance against pathogens with different lifestyles such as *B. cinerea* and *Pst* DC3000, representing necrotrophic and hemibiotrophic pathogens, respectively. It is worthy to note that data from further study on the function of *SlSRN1* in disease resistance against other pathogens will be helpful to understand whether SlSRN1 acts as a global regulator of disease resistance response against different types of pathogens.

ROS accumulated during *B. cinerea* infection has been implicated in susceptible response against necrotrophic fungi like *B. cinerea* [82]. Silencing of *SlSRN1* increased accumulation of ROS in tomato leaves after infection with *B. cinerea* (Fig. 6A and B), which may be due to the changes in activity of superoxide dismutase and catalase induced by *B. cinerea* [83], [84]. Thus, it is likely that silencing of *SlSRN1* promotes the *B. cinerea*-induced accumulation of ROS and thus attenuates disease resistance to this pathogen. On the other hand, expression of *SlPR1a* and *SlPR1b* was induced significantly but expression of *SlPR5* and *SlTPK1b* was reduced markedly in *SlSRN1*-silenced plants after infection of *B. cinerea* (Fig. 6C). The *PR1* gene is mainly regulated through SA-mediated signaling pathway against biotrophic pathogens [85]. The upregulated expression of *SlPR1a* and *SlPR1b* after infection of *B. cinerea,* similar to our previous observation that silencing of *SlMPK4* led to increased expression of *SlPR1s* [86], was observed in some mutants (e.g. Arabidopsis *atwrky33*) with reduced disease resistance to *B. cinerea* [87]. The reduced expression of *SlTPK1b* (Fig. 6C), a regulator of the ethylene (ET)-dependent signaling pathway [72], indicates that silencing of *SlSRN1* attenuates the ET-dependent signaling pathway, which is important for resistance to necrotrophic pathogens [82]. However, increased disease caused by *Pst* DC3000 in *SlSRN1*-silenced plants and induction of *SlSRN1* expression by SA and JA might indicate that SlSRN1 also functions in SA- and/or JA signaling pathways. Thus, it is possible that SlSRN1 positively regulates defense response against different types of pathogens probably through the SA- and JA/ET-mediated signaling pathways. Further study is required to elucidate the molecular mechanism by which SlSRN1 regulates the signaling pathways involved in activation of defense responses against different pathogens.

The involvement of NAC proteins in abiotic stress response has been well documented (for reviews, see [4], [15], [19], [20]). We found in this study that silencing of *SlSRN1* resulted in increased tolerance to oxidative and drought stress (Fig. 7 and 8), indicating that SlSRN1 is a negative regulator of tolerance to oxidative and drought stress. The function of SlSRN1 in oxidative stress tolerance is similar to ANAC016 but contrary to ANAC013 [58], [78]. It was shown that mutation in *ANAC016* resulted in increased tolerance to oxidative stresses [78], whereas overexpression of *ANAC013* increased tolerance to oxidative stress [58]. Previous studies have demonstrated that some membrane-bound NAC proteins including ANAC013 and ANAC017 functions in mitochondrial retrograde regulation (MRR) of the oxidative stress [58], [77]. ROS is thought to be one of the candidate signaling molecules for MRR [58]. The observation that the *SlSRN1*-silenced plants accumulated increased levels of ROS especially H_2_O_2_ after infection by *B. cinerea* (Fig. 6A and 6B) may indicate that silencing of *SlSRN1* potentiate the ability of ROS production upon environmental stress signals. In this regard, the increased level of ROS in the *SlSRN1*-silenced plants during the oxidative stress may signal to initiate MRR and trigger oxidative stress tolerance. It was found that ANAC013 and ANAC017 mediate MRR-induced expression of a set of so-called mitochondrial dysfunction stimulon genes such as *ALTERNATIVE OXIDASE1a* and thus trigger increased oxidative stress tolerance [58], [77]. Furthermore, we found that the leaf discs from the *SlSRN1*-silenced plants had relatively higher level of chlorophyll content than that in the control plants after treatment in H_2_O_2_ (Fig. 7B). This is similar to the observation that mutations in *ANAC016* resulted in delayed leaf senescence (i.e. stay green phenotype) under H_2_O_2_ stress [78]. On the other hand, the function of SlSRN1 in drought stress tolerance as a negative regulator is also opposite to ANAC017, which was shown to be a positive regulator of drought stress tolerance [77]. The difference between SlSRN1 and ANAC017 for their functions in drought stress tolerance might be partially due to different expression patterns and functional diversity of the membrane-bound NAC proteins in different plant species. Expression of *ANAC017* was not induced by stress but mutation in *ANAC017* [77], whereas the expression of *SlSRN1* was induced by drought stress (Fig. 8C and 8D). Although the membrane-bound NAC proteins constitute a specific small group of the NAC family and show a high level of sequence similarity, they seem to have diverse functions in abiotic stress response. For example, ANAC013 and ANAC017 in Arabidopsis have opposite functions in oxidative stress tolerance [58], [77]. Further detailed analysis of gene expression profiling between the *SlSRN1*-silenced and non-silenced plants will be helpful to identify the target genes that are regulated by SlSRN1 and will provide new insights into understanding the mechanism that SlSRN1 regulates abiotic stress tolerance.
